# Aberrant methylation of N-methyl-D-aspartate receptor type 2B (NMDAR2B) in non-small cell carcinoma

**DOI:** 10.1186/1471-2407-11-220

**Published:** 2011-06-05

**Authors:** Hajime Tamura, Makoto Suzuki, Yasumitsu Moriya, Hidehisa Hoshino, Tatsuro Okamoto, Shigetoshi Yoshida, Ichiro Yoshino

**Affiliations:** 1 Department of General Thoracic Surgery, Graduate School of Medicine, Chiba University, 1-8-1 Inohana, Chuoh-Ku, Chiba 280-8670, Japan; 2 Department of Thoracic Surgery, Graduate School of Medicine, Kumamoto University, 1-1-1 Honsou, Kumamoto 860-8556, Japan

## Abstract

**Background:**

N-methyl-D-aspartate receptors (NMDAR) act as tumor suppressors of digestive malignancies. The expression and genetic methylation patterns of *NMDAR2B *in non-small cell lung cancer (NSCLC) are unknown.

**Methods:**

The relationship between gene methylation and expression of *NMDAR2B *was analyzed in NSCLC cell lines (N = 9) and clinical tissues (N = 216). The cell lines were studied using RT-PCR and 5-aza-2'-deoxycytidine treatment, while the clinical tissues were examined by methylation specific real-time quantitative PCR and immunohistochemistry. Retrospective investigation of patient records was used to determine the clinical significance of *NMDAR2B *methylation.

**Results:**

*NMDAR2B *was silenced in five of the nine cell lines; 5-aza-2'-deoxycytidine treatment restored expression, and was inversely correlated with methylation. Aberrant methylation of *NMDAR2B*, detected in 61% (131/216) of clinical NSCLC tissues, was inversely correlated with the status of protein expression in 20 randomly examined tumors. Aberrant methylation was not associated with clinical factors such as gender, age, histological type, or TNM stage. However, aberrant methylation was an independent prognostic factor in squamous cell carcinoma cases.

**Conclusions:**

Aberrant methylation of the *NMDAR2B *gene is a common event in NSCLC. The prognosis was significantly better for cases of squamous cell carcinoma in which *NMDAR2B *was methylated. It may have different roles in different histological types.

## Background

Lung cancer is the leading cause of cancer-related death in Japan as well as in other countries. Although surgical techniques and perioperative managements have progressed, the prognosis of patients with non-small cell lung cancer (NSCLC) remains poor. Various factors are linked to the development of NSCLC, such as smoking tobacco, which is the major risk factor for development of the disease. In addition to identifying the genetic alterations associated with development or progression of NSCLC, other promising avenues of research include finding new molecular therapeutic targets, as that may help improve the survival of patients with this type of cancer.

N-methyl-D-aspartate receptors (NMDAR) are glutamate receptors and constitute the predominant excitatory neurotransmitter receptors in the mammalian brain [1]. NMDAR subunits are also expressed in skeletal muscle, heart muscle, the pancreas [2,3], and suprabasal keratinocytes [4]. NMDARs are heteromeric ligand-gated ion channels that interact with multiple intracellular proteins through different subunits. Among the essential NMDARs (type 2A, type 2B, and type 1), *NMDAR2A *has the greatest structural and functional similarity to *NMDAR2B *[5]. The *NMDAR2A *coding sequence shows 78% homology to human *NMDAR2B *but no significant homology with the human NMDAR1 subunit that is essential for NMDAR function.

The suppressive activities of NMDARs have been documented for a number of processes. For example, NMDAR activation inhibits the outgrowth of keratinocytes necessary for some epithelialization processes. Furthermore, gene expression profiling of human primary glioblastoma multiforme has shown that NMDARs are downregulated in brain tumor samples [4,6]. *NMDAR2B *is methylated in primary human esophagus squamous cell carcinoma (ESCC) tissues and has exhibited tumor-suppressive activity in ESCC cell lines [7]. The status of *NMDAR2B *in NSCLC is unknown.

The present expression and methylation study found that *NMDAR2B *is frequently methylated in NSCLC and that methylation status is associated with some clinical features of NSCLC.

## Methods

### Cell lines and patients

Nine NSCLC cell lines (HCC193, HCC366, HCC515, HCC1171, NCI-H1395, NCI-H1770, NCI-H1993, NCI-H2126, and NCI-H2882) were examined in this study. These cell lines were established and kindly provided by Dr. Adi Gazdar of the University of Texas Southwestern Medical Center. Cell cultures were grown in RPMI-1640 medium (Life Technologies, Inc., Rockville, MD, USA) supplemented with 5% CO_2 _at 37°C. Normal bronchial epithelial cells (NHBECs) were cultured as reported previously [8] and normal trachea RNA was obtained from Clontech (Palo Alto, CA, USA).

Surgically resected tumor samples and 120 non-malignant lung tissues were obtained from 216 unselected NSCLC patients who had not received any treatment prior to resection at the Chiba University Hospital, Chiba, Japan, from 1995 to 2000. NSCLC include adenocarcinoma (N = 116), squamous cell carcinoma (N = 76), large cell carcinoma (N = 12), and adeno-squamous cell carcinoma (N = 2). This study was approved by the Institutional Review Board, and written informed consent was obtained from each participant. All patients received curative intent surgery. Resected samples were immediately frozen and stored at -80°C until used. Methylation status was determined for each patient sample, and 20 of the 216 cases were randomly selected for immunohistochemical examination to compare expression and methylation in primary samples.

### RNA preparation and Reverse Transcriptase-PCR

RNA was extracted using Trizol (Invitrogen, Carlsbad, CA, USA) and reverse transcribed with SuperScript II reverse transcriptase (Invitrogen). *GAPDH *expression was used as an internal control to confirm the success of the reverse transcription reaction. The primer sequences used for reverse transcription of *NMDAR2B *and *GAPDH *are shown in Table 1. PCR products were analyzed on 2% agarose gels stained with ethidium bromide. NHBEC and normal trachea were used as normal controls for reverse transcriptase-PCR (RT-PCR).

**Table 1:** Primer and probe sequences

Primer	Assay	Sequence
	
NMDAR2B	RT-PCR	5'-GCCTGAGCGACAAAAAGTTC-3' (forward)
NMDAR2B	RT-PCR	5'-CATCTCCCCATCTCCAAAGA-3' (reverse)

GAPDH	RT-PCR	5'-CACTGGCGTCTTCACCACCATG-3' (forward)

GAPDH	RT-PCR	5'-GCTTCACCACCTTCTTGATGTCA-3' (reverse)

NMDAR2B TAQF	Taqman-MSP	5'-GAGTATGGTTATTTTTAAAGCG-3'

NMDAR2B TAQR	Taqman-MSP	5'-TTAAAACGAATTAATATCTTTTTCG-3'

NMDAR2B probe	Taqman-MSP	6FAM5'-ATTCGCGTGTTTTTCGGAGGGTGA-3' TAMRA

β-actin TAQF	Taqman-MSP	5'-TGGTGATGGAGGAGGTTTAGTAGTAAGT-3'

β-actin TAQR	Taqman-MSP	5'-AACCAATAAAACCTACTCCTCCCTTAA-3'

β-actin probe	Taqman-MSP	6FAM5'-ACCACCACCCAACACACAATAACAAACACA-3' TAMRA

### Treatment of tumor cell lines with 5-aza-2'-deoxycytidine

Five tumor cell lines with negative gene expression were incubated in culture medium with 1 μM of the demethylating agent 5-aza-2'-deoxycytidine (Sigma-Aldrich, St. Louis, MO, USA) for six days, with medium changes on days one, three, and five. Cells were harvested and RNA was extracted on day six.

### DNA preparation and real-time quantitative PCR

Genomic DNA was obtained from lung cancer cell lines, cultured nonmalignant cells, primary tumors and adjacent nonmalignant tissues after overnight digestion with sodium dodecyl sulfate and Proteinase K (Life Technologies, Inc.) at 37°C, followed by standard phenol chloroform (1:1) extraction and ethanol precipitation.

**Figure 1:**
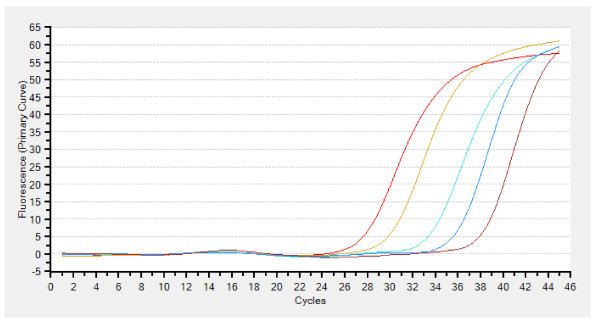
**Calibration curve**. Serial dilutions of human leukocyte genomic DNA, which was methylated *in vitro*, were used to construct a calibration curve.

DNA was treated with sodium bisulfite as described previously [9]. DNA methylation patterns in the CpG island of the gene were determined using methylation specific real-time quantitative PCR (Taqman-MSP) [7]. All oligonucleotide primer pairs were purchased from Invitrogen, while Taqman probes were purchased from VWR (West Chester, PA, USA). Taqman primer and probe sequences for *NMDAR2B *and *β-actin *are listed in Table 1. Serial dilutions of human leukocyte genomic DNA, which was methylated *in vitro*, were used to construct a calibration curve (Figure 1) (Thermal Cycler Dice^® ^Real Time System TP 800); all reactions were performed in duplicate. The methylation ratio was defined as the quantity of fluorescence intensity derived from NMDAR2B promoter amplification divided by fluorescence intensity from β-actin amplification and multiplied by 100 [Taqman methylation value (TaqMeth V)]. Cut off value was defined as 1.0, based on previous reports [7].

### Immunohistochemistry (IHC)

Four micron sections were used for the tissue arrays. Tissue arrays were deparaffinized in xylene, rehydrated in graded alcohol, and transferred to PBS. Thereafter, antigen retrieval was carried out via a microwave in 0.01 M citrate buffer, pH 6.0. Endogenous peroxidase activity was blocked by incubating sections in hydrogen peroxide (0.3%, v/v) for 15 min. Non-specific binding was blocked with 1% (w/v) BSA in PBS for one hr followed by incubation with anti-NMDAR2B polyclonal antibody (1:100) (Chemicon, Temecula, CA, USA) for 16 hr at 4°C. The primary antibody was detected using biotinylated secondary antibody and peroxidase-labeled streptavidin complex using the Dako LSAB Plus Kit (Dako, Glostrup, Denmark). Color was developed using the chromogen, diaminobenzidine (DAB). Finally, the slides were counter stained with Mayer's hematoxylin and mounted with D.P.X. Parallel sections in which the primary antibody was replaced by nonimmune rabbit IgG of the same isotype were examined to ensure specificity and exclude cross reactivity between the antibodies and conjugates used (negative control).

The staining intensity of NMDAR2B expression was graded semi-quantitatively into none, weak, moderate, and strong. Overexpression of NMDAR2B was considered positive if > 30% of tumor cells were moderately or strongly stained. Slides were blindly evaluated at three different times and the average levels were used by two pathologists (H.T. and Y.M.) for statistical analyses. IHC photoimaging was done by Carl Zeiss MicroImaging GmbH and a digital camera (Canon Power Shot A640).

### Statistical Analysis

Fisher's exact tests were used to assess the association between categorical variables. Overall survival curves were calculated with the Kaplan-Meier method and were compared using the log-rank test. The Cox proportional hazards regression model was used for multivariate analysis. Statistical significance was defined as *P *values less than 0.05.

## Results

### Expression and methylation analysis in NSCLC cell lines

**Figure 2:**
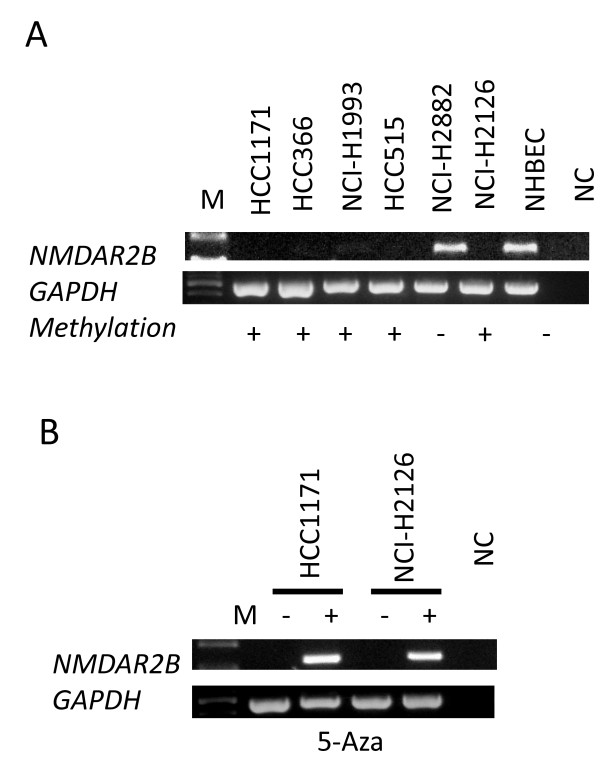
**Expression and methylation of *NMDAR2B *in NSCLCs cell lines**. Representative examples of reverse transcriptase-polymerase chain reaction results for N-methyl-D-aspartate receptor type 2B (*NMDAR2B*) expression in lung cancer cell lines (A), and the effect of 5-aza-2'-deoxycytidine on *NMDAR2B*-negative cell lines (B). Treatment with 5-Aza-CdR restored the expression of *NMDAR2B *in four cell lines. Expression of the housekeeping gene glyceraldehyde-3-phosphate dehydrogenase (*GAPDH*) was run as a control for RNA integrity. M indicates size marker; NC, negative control (water blank); -, before 5-Aza-CdR treatment; +, after 5-Aza-CdR treatment.

The gene expression levels of *NMDAR2B *in NSCLC cell lines were assessed by RT-PCR (Figure 2). A comparison between tumor cell lines and NHBEC and trachea cells showed that five out of nine cell lines had lost *NMDAR2B *expression (Table 2). The non-expressing cell lines were treated with 5-aza-2'-deoxycytidine (5-Aza-CdR) to confirm that aberrant methylation was responsible for silencing *NMDAR2B *expression. *NMDAR2B *expression was upregulated by 5-Aza-CdR in five out of five lines (Figure 2, Table 2). Thus, aberrant methylation was found in five out of nine NSCLC cell lines and was inversely correlated with *NMDAR2B *expression (Table 2).

**Table 2:** Expression and aberrant methylation profiles of *NMDAR2B *in NSCLC cell lines

	Taqman-MSP	RT-PCR	5-Aza
HCC193	-	+	ND*
HCC366	+	-	+
HCC515	+	-	+
HCC1171	+	-	+
NCI-H1395	-	+	ND
NCI-H1770	-	+	ND
NCI-H1993	+	-	+
NCI-H2126	+	-	+
NCI-H2882	-	+	ND

* Not done

### Aberrant methylation of *NMDAR2B *in clinical NSCLC tissues

**Figure 3:**
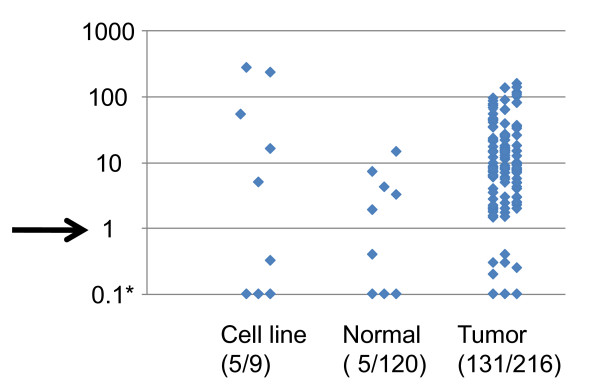
**Quantitative analysis of *NMDAR2B *gene expression in NSCLCs**. Taqman-MSP analysis with a probe targeted to the CPG island of *NMDAR2B *was performed using 120 non-malignant lung tissues, 216 NSCLC samples, and nine cell lines. The cut-off value was defined as 1.0 based on previous reports [7]; five of 120 nonmalignant lung tissue samples were negative by this cut-off value.

*NMDAR2B *methylation in primary tumors and non-malignant tissues was analyzed by Taqman MSP (Figure 3). Of 216 NSCLCs, 131 (61%) were methylated, while 5 (4%) of 120 corresponding non-malignant lung tissues were methylated, indicating that *NMDAR2B *methylation was a tumor-specific event (*P *< 0.0001).

**Figure 4:**
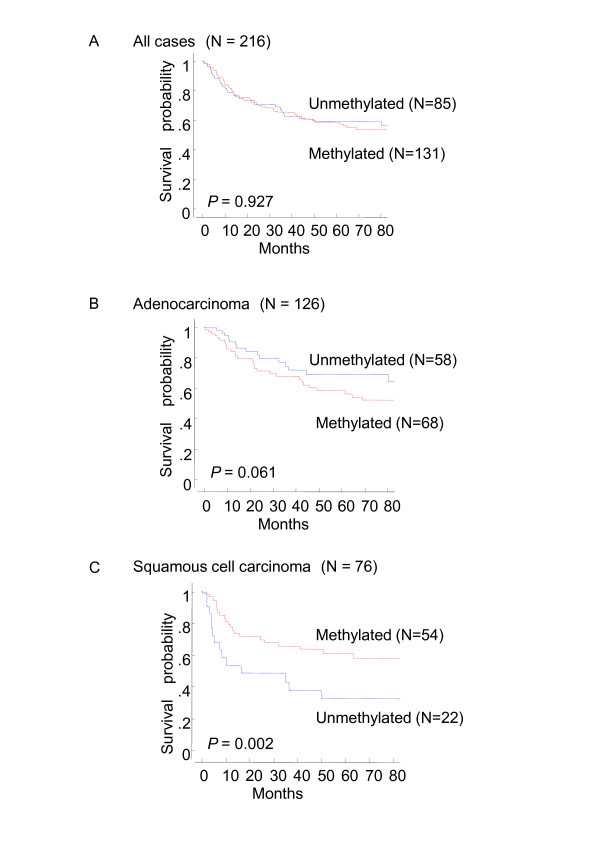
**Survival of NSCLC patients and *NMDAR2B *gene methylation status**. Kaplan-Meier curves of overall survival for 216 patients with lung cancer (A), 126 patients with adenocarcinoma (B) and 76 patients with squamous cell carcinoma (C). Patients with squamous cell carcinoma who had aberrantly methylated *NMDAR2B *genes had a significantly better survival rate compared with patients with unmethylated receptor genes (*P *= 0.002).

The relationship between methylation and clinical features was examined. No significant associations among gender, age, smoking, histological type, or stage were observed (Table 3). In terms of survival, the presence of *NMDAR2B *methylation was significantly associated with a better prognosis in squamous cell carcinoma cases (*P *= 0.002), but not in adenocarcinoma cases (Figure 4). Cox proportional hazard regression analysis determined that *NMDAR2B *methylation is a prognostic factor independent of TNM staging (Table 4). Prognosis was significantly better for cases of squamous cell carcinoma in which *NMDAR2B *was methylated.

**Table 3:** Methylation status of *NMDAR2B *and clinicopathologic factors in clinical NSCLCs

Cases (N = 216)		Methylation (N = 131, 61%)
Gender	Male (152)	93 (61) *
	Female (64)	38 (59)
Age**	< 65 (107)	62 (58)
	≥ 65 (109)	69 (63)
Smoking	Never (64)	36 (56)
	Smoker (152)	95 (63)
Histology	Adenocarcinoma (126)	68 (54)
	Others*** (90)	63 (70)
T factor	T1 (135)	81 (60)
	T2, 3, 4 (81)	50 (62)
N factor	N0 (115)	73 (63)
	N1, 2, 3 (101)	58 (57)
Stage	I (80)	48 (60)
	II, III, IV (136)	83 (61)

*Parentheses in each column indicate percentage of methylation

**Divided by median age

***Others include squamous cell, adenosquamous cell, and large cell carcinoma

**Table 4:** Multivariate analyses of prognostic variables in patients with squamous cell carcinoma of the lung

Variable	Multivariate		
	Hazard rate	95%CI*	*P*
Gender(male/female)	0.514	0.20-1.32	0.1666
Age	1.011	0.973-1.050	0.586
Lymph node metastasis -/+	2.257	1.136-4.464	0.02
*NMDAR2B *methylation -/+	0.381	0.188-0.775	0.0077
Gender(male/female)	0.474	0.183-1.228	0.1241
Age	1.012	0.975-1.051	0.5196
Stage I, II/III, IV	2.096	0.971-4.525	0.0596
*NMDAR2B *methylation -/+	0.336	0.165-0.684	0.0026

* Confidence Interval

### Expression of NMDAR2B protein in primary tumors

**Figure 5:**
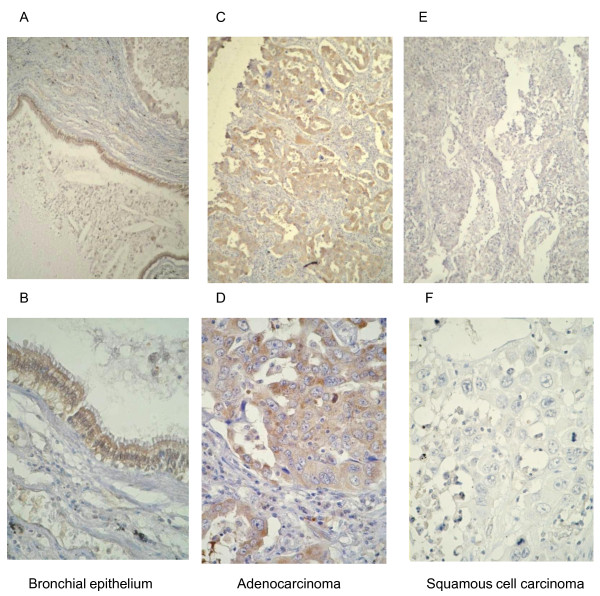
**Immunohistochemical staining of NMDAR2B in NSCLC tissue**. NMDAR2B expression in bronchial epithelium (A, B; positive expression), adenocarcinoma (C, D; positive expression) and squamous cell carcinoma (E, F; low expression).

The typical immunostaining pattern for NMDAR2B in NSCLC is shown in Figure 5. NMDAR2B was expressed in bronchial epithelial cells. Using the criteria described in the Methods section, low NMDAR2B expression was found in 11 of 20 (55%) tumors, while moderate to strong expression was found in nine (45%) tumors. In eight of the 11 cases, there was low expression of NMDAR2B protein and methylation of *NMDAR2B*. The remaining nine cases exhibited moderate to strong expression of NMDAR2B and unmethylated *NMDAR2B*. Thus, there was a significant inverse correlation between the DNA methylation status of *NMDAR2B*and the level of protein expression (*P *= 0.0014).

## Discussion

Kim et al. demonstrated down-regulation of *NMDAR2B *in esophageal cancer cells through aberrant methylation [7]. In this study, decreased *NMDAR2B *expression in NSCLC cells was associated with aberrant methylation of the gene. Expression was restored by treating NSCLC cells with a demethylating agent. Although there are other possible mechanisms for down-regulation of *NMDAR2B *expression, the excellent concordance between mRNA expression (RT-PCR) and protein expression (IHC), indicates that down-regulation in NSCLC occurs primarily through aberrant methylation.

Among novel methylation genes, *NMDAR2B *was found to have a high frequency of methylation in primary ESCC and strong apoptotic activity in ESCC and gastric cell lines [7,10]. Studies of NMDAR signaling have been conducted and the role of NMDAR activity in apoptosis has been studied intensively in neurons [11,12], as well as its functional role as a tumor suppressor in human tumors. NMDAR-mediated apoptosis in human ESCC cell lines was blocked by a specific NMDAR2B inhibitor, ifenprodil. Surprisingly, BAPRA-AM, a calcium chelator, was unable to protect cells against NMDAR2B-induced apoptosis. These results contrast with previous work in neurons, which indicated that functional reconstitution of NMDAR-induced apoptosis in NSCLC took place through a Ca^2 ^^+ ^permeability independent mechanism [7]. However, the specific mechanism underlying the growth suppressive effects and the larger role of *NMDAR2B *inactivation in the development of NSCLCs are unknown.

NMDARs constitute the predominant excitatory neurotransmitter receptors in the mammalian brain. Therefore, the relationship between NMDARs and neuroendocrine tumors is of interest. Neuroendocrine tumors in lung cancer exist among small cell lung carcinomas (SCLC) and large cell carcinomas. Further study will be needed to distinguish between *NMDAR2B *methylation and neuroendocrine tumors such as SCLC or large cell carcinoma with neuroendocrine differentiation.

The significance of NMDAR2B methylation in the survival of patients with squamous cell carcinoma was analyzed here by a log-rank test and the Cox proportional hazards regression model. However, due to the relatively small size of the cohorts in this study (squamous cell carcinoma, N = 76), further validation is required.

This study shows that *NMDAR2B *in lung squamous cell carcinomas may be associated with favorable prognoses. There are few reports regarding disease prognosis and gene methylation in lung squamous cell carcinoma [13-16]. The clinical outcome among patients with lung squamous cell carcinoma is improved by Gemcitabine [17], while Pemetrexed is particularly active in non-squamous NSCLC histology [18], and it is now clear that chemosensitivity differs according to histology. Thus, the correlation between chemosensitivity and squamous cell carcinoma histology is now being analyzed, and questions remain. For example is there a correlation between chemosensitivity and *NMDAR2B *methylation? Is *NMDAR2B *methylation a better predictive marker than tumor histology? A potential link between NMDAR2B methylation in squamous cell carcinoma and subtype-specific chemosensitity has to be investigated in future studies. Further studies are needed to determine the utility of *NMDAR2B *methylation and its correlation to chemosensitivity, as a predictive marker in squamous cell carcinoma or other cell types.

## Conclusions

The results suggest that *NMDAR2B *methylation is closely correlated with decreased or absent expression in NSCLC. Because NMDAR2B methylation is common and specific in NSCLC, it may serve as an important molecular marker, especially in squamous cell carcinomas.

## Abbreviations

*NMDAR2B*: N-methyl-D-aspartate receptor type2B; *NSCLC*: non-small cell lung cancer; *ESCC*: Esophagus squamous cell carcinoma; *NHBECs*: Normal bronchial epithelial cells; *5-Aza-CdR*: 5-aza-2'-deoxycytidine; *Taqman-MSP*: methylation specific real-time quantitative PCR; *IHC*: immunohistochemistry; *SCLC*: small cell lung carcinoma;

## Competing interests

The authors declare that they have no competing interests.

## Authors' contributions

HT and MS designed cellular and molecular experiments, performed cellular and molecular experiments, and drafted the manuscript. YM, HH, TO, and SY participated in the design of the study and revised the manuscript. IY participated in the overall design and study coordination, and finalized the draft of the manuscript. All authors read and approved the final manuscript.

## Acknowledgements

This work was supported by a grant from the Smoking Research Foundation (2010).

## Pre-publication history

The pre-publication history for this paper can be accessed here:

http://www.biomedcentral.com/1471-2407/11/220/prepub
